# Two essential MYST-family proteins display distinct roles in histone H4K10 acetylation and telomeric silencing in trypanosomes

**DOI:** 10.1111/j.1365-2958.2008.06346.x

**Published:** 2008-07-09

**Authors:** Taemi Kawahara, T Nicolai Siegel, Alexandra K Ingram, Sam Alsford, George A M Cross, David Horn

**Affiliations:** 1 London School of Hygiene and Tropical MedicineKeppel Street, London WC1E 7HT, UK; 2 Laboratory of Molecular Parasitology, The Rockefeller UniversityNew York, NY 10065, USA

## Abstract

Chromatin modification is important for virtually all aspects of DNA metabolism but little is known about the consequences of such modification in trypanosomatids, early branching protozoa of significant medical and veterinary importance. MYST-family histone acetyltransferases in other species function in transcription regulation, DNA replication, recombination and repair. *Trypanosoma brucei* HAT3 was recently shown to acetylate histone H4K4 and we now report characterization of all three *T. brucei* MYST acetyltransferases (HAT1–3). First, GFP-tagged HAT1–3 all localize to the trypanosome nucleus. While HAT3 is dispensable, both HAT1 and HAT2 are essential for growth. Strains with HAT1 knock-down display mitosis without nuclear DNA replication and also specific de-repression of a telomeric reporter gene, a rare example of transcription control in an organism with widespread and constitutive polycistronic transcription. Finally, we show that HAT2 is responsible for H4K10 acetylation. By analogy to the situation in *Saccharomyces cerevisiae*, we discuss low-level redundancy of acetyltransferase function in *T. brucei* and suggest that two MYST-family acetyltransferases are essential due to the absence of a Gcn5 homologue. The results are also consistent with the idea that HAT1 contributes to establishing boundaries between transcriptionally active and repressed telomeric domains in *T. brucei*.

## Introduction

Histone N-terminal tails extend from the core of the nucleosome and can be post-translationally modified with a range of chemical groups. These combined modifications may constitute a code (Jenuwein and Allis, 2001) that regulates access to DNA, possibly during all chromatin-templated processes (Millar and Grunstein, 2006). For example, the transfer of an acetyl group from Ac-CoA to specific Lys ε-amino groups on histone H4 can modulate chromosome structure and interaction (Shogren-Knaak *et al*., 2006) or provide binding platforms for regulatory factors (de la Cruz *et al*., 2005). For identical DNA sequences to exhibit distinct properties within chromatin, cells are equipped with the enzymes that specifically and reversibly modify the histones in this way. Acetylation of histones H3 and H4 has been characterized in some detail in *Saccharomyces cerevisiae* and in human cells (Rice and Allis, 2001; Lachner and Jenuwein, 2002) but comparison among organisms ‘argue against there being a universal histone code and underscore the need to avoid general conclusions obtained from one organism’ (Garcia *et al*., 2007).

The MYST family is a group of related histone acetyltransferases that appears to have members in all eukaryotes (Yang, 2004). The acronym MYST is from the founding members, human MOZ (monocytic leukaemia zinc finger protein) (Borrow *et al*., 1996), yeast Ybf2 (renamed Sas3, for Something about silencing 3) and Sas2 (Reifsnyder *et al*., 1996) and mammalian TIP60 (HIV Tat-interacting protein 60 kDa) (Kamine *et al*., 1996). A third MYST protein in *S. cerevisiae* is Esa1 (Essential sas2-related acetyltransferase 1) (Smith *et al*., 1998; Clarke *et al*., 1999). Besides MOZ and TIP60 in humans are hMOF (orthologue of *Drosophila* Mof, males-absent-on-the-first) (Taipale *et al*., 2005), HBO1 (HAT bound to ORC1, replication origin recognition complex 1) (Iizuka and Stillman, 1999) and MORF (MOZ-related factor) (Champagne *et al*., 1999).

MYST acetyltransferases have been shown to impact upon transcription, DNA replication, recombination and repair (Carrozza *et al*., 2003). The enzymes function in multi-protein complexes *in vivo* (Lee and Workman, 2007). For example, *S. cerevisiae* Esa1, Sas2 and Sas3 are the catalytic components of the NuA4 (Nucleosomal H2A/H4) (Eisen *et al*., 2001; Doyon and Cote, 2004), SAS (Sutton *et al*., 2003) and NuA3 (Nucleosomal Acetyltransferase of histone H3) (John *et al*., 2000) complexes respectively. Other components of the complexes may be required for activity (Sutton *et al*., 2003) or substrate recruitment (Lee and Workman, 2007) while there is also an interplay between histone acetylation and methylation. Indeed, MYST acetyltransferase complexes may require histone methylation for substrate recruitment (Martin *et al*., 2006) and MYST acetyltransferases can function in the same complex as a methyltransferase (Dou *et al*., 2005).

Trypanosomatids, including *Trypanosoma brucei*, *T. cruzi* and *Leishmania* spp., branched early in the eukaryotic lineage and trypanosomes are established, ‘differently evolved’ model organisms. Although trypanosome histone tails are divergent, acetylation has been identified on histone H4K2 (2% of sites modified), K4 (73%), K5 (7%), K10 (7%) and K14 (1%) (da Cunha *et al*., 2006; Janzen *et al*., 2006a; Mandava *et al*., 2007). These mono-flagellated protozoa have a devastating impact on the world's poor, causing African trypanosomiasis, Chagas disease and leishmaniasis (http://www.who.int/tdr/diseases/). The consequences of this range of human and animal diseases are hundreds of thousands of deaths each year, ∼1.5 million cases a year of the disfiguring lesions associated with cutaneous leishmaniasis and severely curtailed agricultural development throughout sub-Saharan Africa. Human African trypanosomaiasis is fatal if untreated and represents the leading cause of mortality in some areas. *T. brucei* also causes Nagana in cattle, rendering 10 million square kilometres of land unsuitable for livestock.

In trypanosomatids, almost all genes are transcribed as long polycistrons (Clayton, 2002). No RNA polymerase II (pol II) promoters have been identified for protein-coding genes but certain protein-coding genes are transcribed by RNA polymerase I (pol I) (Palenchar and Bellofatto, 2006). Although gene expression in trypanosomatids is predominantly regulated post-transcriptionally (Clayton, 2002; Horn, 2008), several lines of evidence point to important roles for chromatin structure and modification in gene expression, cell cycle control and differentiation (Ersfeld *et al*., 1999; Belli, 2000; Horn, 2001). More recently, acetylated and methylated histones were shown to be enriched at regions where the polycistronic transcription units diverge in *T. cruzi* (Respuela *et al*., 2008). One of the four putative class I/II histone deacetylases characterized in *T. brucei* is required for normal cell cycle progression (Ingram and Horn, 2002) while the only nuclear class III, sirtuin-type deacetylase (Kowieski *et al*., 2008) is involved in DNA repair (Garcia-Salcedo *et al*., 2003) and telomeric gene silencing (Alsford *et al*., 2007). Trypanosomatid genome sequencing revealed six putative histone acetyltransferases in *T. brucei*: three related to the MYST family, two of the elongator type (see table S4 in Ivens *et al*., 2005) and a PHD-finger protein (D. Horn, unpublished). HAT3, one of the MYST-family proteins, was recently shown to be the histone H4K4 acetyltransferase (Siegel *et al*., 2008). We now report characterization of all three MYST-family proteins (HAT1–3). While HAT3, the H4K4 acetyltransferase, is dispensable for growth, HAT1 is required for telomeric silencing and growth, and possibly, for DNA replication and HAT2 is required for H4K10 acetylation and growth.

## Results

### Three MYST-family acetyltransferases expressed in *T. brucei*

**Fig. 1:**
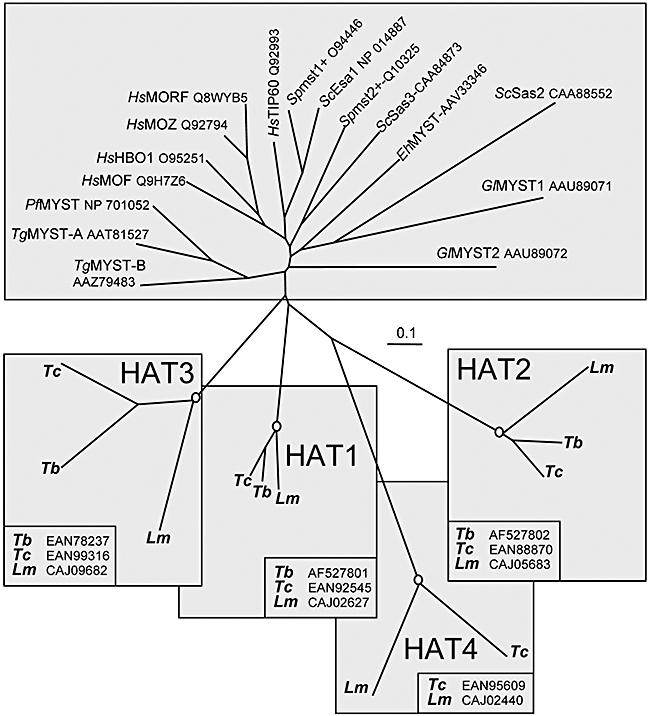
Phylogenetic analysis. The trypanosomatid HATs were compared with other MYST-family proteins. The unrooted neighbour-joining tree was generated using clustal 1.8X and TreeView. Where excellent (≥ 99.9%), branching confidence is indicated (open circles). *Tb*, *T. brucei*; *Tc*, *T. cruzi*; *Lm*, *L. major*; *Hs*, *Homo sapiens*; *Sc*, *S. cerevisiae; Gl*, *Giardia lamblia*; *Eh*, *Entamoeba histolytica*; *Sp*, *Schizosaccharomyces pombe*; *Pf*, *Plasmodium falciparum*; *Tg*, *Toxoplasma gondii*. All accession numbers are indicated. The GeneDB IDs for the *T. brucei* proteins are: HAT1, Tb927.7.4560; HAT2, Tb11.01.3380; and HAT3, Tb10.6k15.2190.

Four proteins were identified encoded by *T. cruzi* and *Leishmania major* and three by *T. brucei* related to the MYST-family acetyltransferases (Ivens *et al*., 2005). For comparison, the human genome encodes five MYST-family proteins: *S. cerevisiae*, three and *Schizosaccharomyces pombe*, two (Yang, 2004). The trypanosomatid proteins were designated HAT1–4 for putative Histone AcetylTransferase and it is *HAT4* that is not present in the corresponding syntenic region or elsewhere in the *T. brucei* genome. Phylogenetic analysis indicated that the trypanosomatid proteins are remarkably divergent relative to other MYST-family members (Fig. 1). Trypanosomatid MYST proteins cluster into distinct groups but can all be traced to the root of the tree through a branch not shared with any other species. This suggests that the MYST proteins may have diversified in a common trypanosomatid ancestor. Thus, shared phylogeny does not allow us to identify putative homologues for the trypanosomatid proteins in model organisms.

**Fig. 2:**
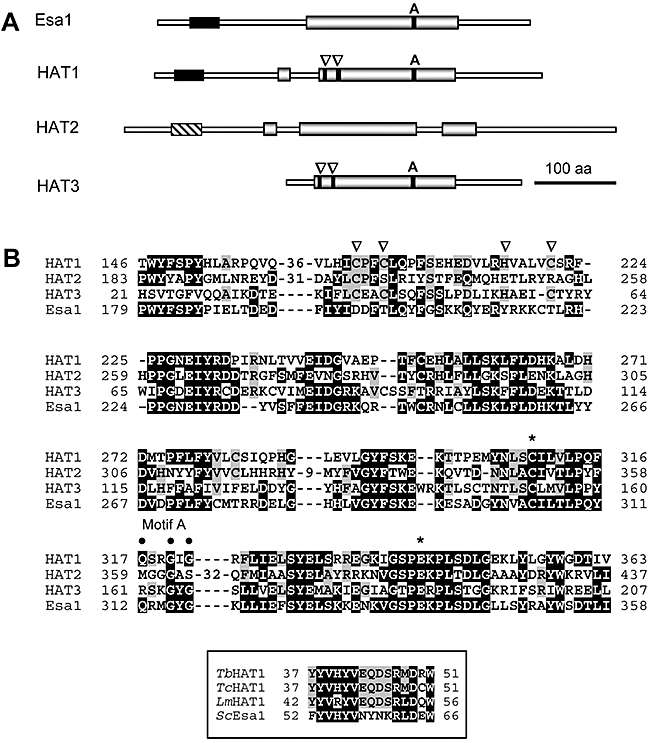
MYST-family acetyltransferases in *T. brucei*. A. Schematic representation of the predicted *T. brucei* MYST-family proteins compared with *S. cerevisiae* Esa1. The core acetyltransferase domains (grey boxes) and N-terminal chromodomains (black boxes and cross-hatched box; see the text) are indicated. Arrowheads indicate a C_2_HC zinc-finger motif. ‘A’ indicates motif A. B. Sequence alignment. The core acetyltransferase domains from the *T. brucei* HATs were aligned with the equivalent region from Esa1 using clustalw followed by manual adjustment. Residues that are shared between Esa1 and any of the *T. brucei* proteins are white on a black background. Other residues shared among the *T. brucei* proteins are on a grey background. Motif A and the zinc-finger motif are indicated (see A). Asterisks indicate a glutamate residue [E] and a cysteine residue [C] required for Esa1 catalytic activity. Insertions within the HAT1 and HAT2 MYST-homology domains have been removed to optimize the alignment. The box shows the trypanosomatid chromodomain from HAT1 aligned with the corresponding domain from *S. cerevisiae* Esa1.

A schematic representation of the *T. brucei* MYST proteins is shown in Fig. 2A. Alignment and sequence comparison revealed a number of features (see Fig. 2B). Motif A (Q/Rx_2_GxG/A) is involved in binding Coenzyme A (Roth *et al*., 2001) and this motif is present in HAT1 (Qx_2_GxG in all three trypanosomatids) and HAT3 (Rx_2_GxG in all three trypanosomatids) and can also be found in HAT4 in *T. cruzi* and *Leishmania* (not shown). Key residues found to be critical for catalytic activity in Esa1 are Glu^338^ and Cys^304^ (Yan *et al*., 2000; 2002) and these strictly conserved residues are also found in HAT1–3 in all three trypanosomatids. In Esa1, these residues participate in a ping-pong catalytic mechanism involving a Cys^304^ self-acetylation intermediate (Yan *et al*., 2002). Like several MYST-type acetyltransferases, HAT1 and HAT3 in all three trypanosomatids (see *T. brucei* proteins in Fig. 2) and HAT4 in *T. cruzi* and *Leishmania* have a C_2_HC (Cx_2_Cx_12_Hx_3−5_C) zinc-binding motif thought to be involved in catalytic activity and/or substrate recognition (Akhtar and Becker, 2001). Motif A and the C_2_HC motif are absent from all three trypanosomatid HAT2s. Relative to Esa1, HAT1 and HAT2 have insertions within the MYST-homology domain (one in HAT1 and two in HAT2, see Fig. 2) and similarly located, but variable-size, insertions are also found in these proteins in *T. cruzi* and *Leishmania*. Several MYST-family proteins contain an N-terminal chromo (chromosome organization modifier) domain thought to deliver these regulators to their sites of action on chromatin (Jones *et al*., 2000) by mediating binding to methyl-lysine (Nielsen *et al*., 2002). The chromodomain of HP1 (heterochromatin protein 1) recognizes histone H3 methylated on Lys^9^ for example (Bannister *et al*., 2001) but these domains may also interact with RNA (Akhtar *et al*., 2000). Sequence alignments suggest the presence of chromodomains towards the N-terminus of both HAT1 and HAT2. The evidence is weaker for HAT2, but HAT1 alignment with Esa1 (Fig. 2A, boxed) indicates the presence of a MOF-like chromo-barrel domain (Nielsen *et al*., 2005) suggesting a link between acetylation and methylation in trypanosomatids. Taken together, our analysis indicates that each of the HATs has similar features regardless of the trypanosomatid under consideration. We have focused on the three *T. brucei* HATs (1–3) found on chromosomes 7, 11 and 10, encoding proteins with predicted molecular mass of approximately 53.5, 67.7 and 32.4 kDa respectively.

We analysed expression and mapped RNA processing signals using reverse transcription polymerase chain reaction (RT-PCR). This technique exploits the fact that all mature *T. brucei* mRNAs possess an identical 5′-spliced leader (SL) sequence added at a specific AG dinucleotide, splice acceptor. DNA sequencing identified acceptor sites 84, 64 and 5 nt upstream of the start codons for *HAT1–3* respectively. Using Northern blotting, we detected cognate mRNA transcripts for HAT1–3 of approximately 2.1 kb, 2.1 kb and 1.3 kb respectively. All three genes are expressed in bloodstream and insect-stage cells, and mRNA abundance revealed no evidence for differential expression in these major life cycle stages (data not shown).

### All three acetyltransferases localize to the *T. brucei* nucleus

**Fig. 3:**
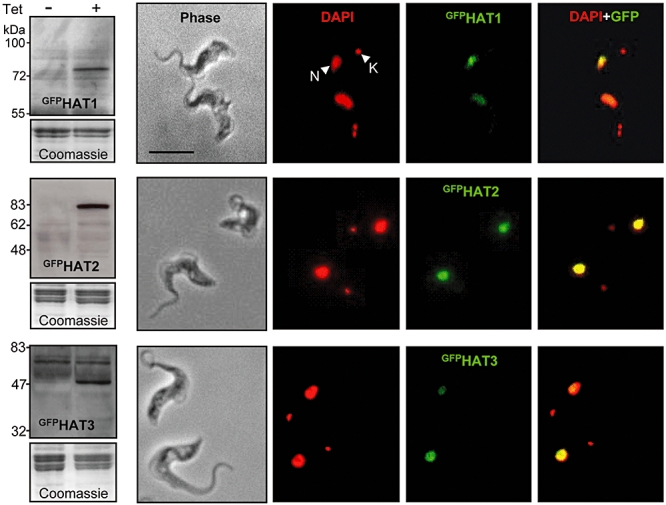
MYST-family proteins localize to the *T. brucei* nucleus. Strains were cultured without Tet (−) or with Tet (1 μg ml^−1^) for 24 h (+) to induce ^GFP^HAT expression. Fusion proteins were detected by Western blotting using α-GFP (left). Coomassie-stained gels are shown as loading controls. Representative examples of immunofluorescence (HAT1 and HAT3) or direct fluorescence (HAT2) images of induced cultures are shown. HAT localization is indicated in green. DAPI, the DNA counterstain (4′,6-diamino-2-phenylindole), is false-coloured red and reveals the nucleus (N) and the smaller mitochondrial, kinetoplast DNA (K). Merged images indicate nuclear colocalization in yellow. Scale bar: 10 μm.

Eukaryotic MYST acetyltransferases typically localize to the nucleus. Nuclear localization signals are not well defined in trypanosomatids and we obtained no clear prediction for subcellular localization from bioinformatic analysis so we determined subcellular localization in bloodstream-form strains engineered for Tet-inducible expression (Alsford *et al*., 2005) of GFP-tagged versions of each gene. ^GFP^HAT1–3 peptides are predicted to be 80.6, 94.7 and 59.5 kDa, respectively, and proteins of the predicted size were conditionally expressed in each strain as demonstrated by Western blotting (Fig. 3). Microscopic analysis of these strains did not reveal any significant GFP signal in un-induced cultures (data not shown). In contrast, fluorescence or immunofluorescence analysis of each induced ^GFP^HAT strain indicated specific accumulation in the nucleus (Fig. 3). These results were confirmed with strains expressing epitope (myc)-tagged versions of each acetyltransferase (data not shown).

### HAT3 is dispensable while HAT1 and HAT2 are essential for growth

**Fig. 4:**
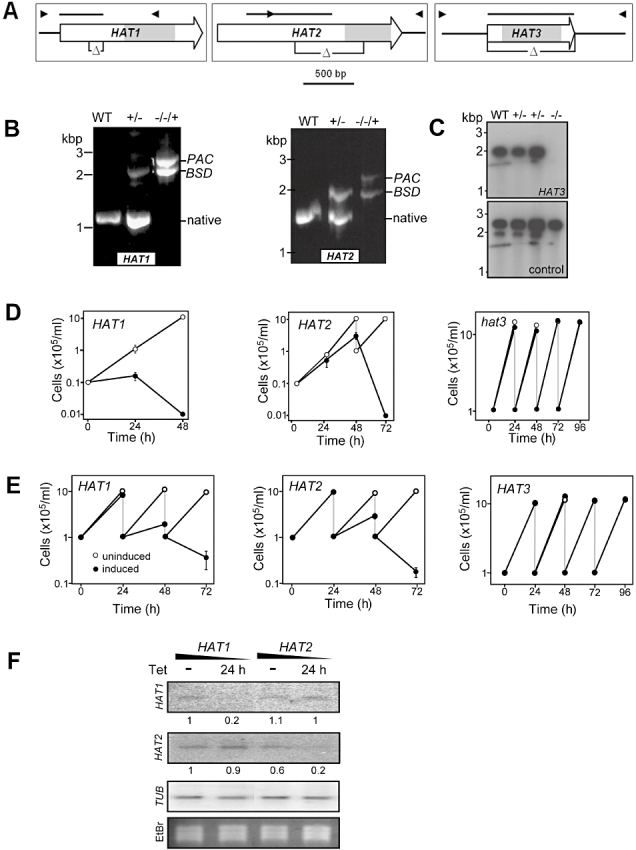
*HAT3* is dispensable while *HAT1* and *HAT2* are essential for growth. A. Schematics illustrating the gene targeting strategies. The symbols ‘Δ’ indicate regions targeted for deletion. Arrowheads represent the positions of the primers used for PCR assays; 29M45/3, 18M145/3 and H31/4 for *HAT1–3* respectively; bars represent probes used for Southern and Northern blot analysis and the grey boxes indicate the regions targeted for RNAi by dsRNA. B. Genomic DNA from clones with both native alleles disrupted in the presence of conditionally expressed ^GFP^HAT1 and ^GFP^HAT2 were analysed by PCR and the products were visualized in agarose gels. The *BSD* and *PAC* cassettes are 1 and 1.3 kb respectively. The recombinant ^GFP^HAT genes are not detected using this assay (see primer locations in A). C. Southern blot analysis of *hat3* null *T. brucei*. Genomic DNA was digested with AccI and the blot was sequentially hybridized with the probes indicated (the control probe is from HAT1, see bars in A). D. Growth analysis of strains expressing conditional copies of ^*GFP*^*HAT1* or ^*GFP*^*HAT2* and a *hat3* null strain. For ^*GFP*^*HAT1* and ^*GFP*^*HAT2*: ^*GFP*^*HAT* expressed (+Tet), open circles; ^*GFP*^*HAT* expression inactivated (−Tet), closed circles. For *HAT3*: wild type, open circles; *hat3* null, closed circles. Cells were split back to 1 × 10^5^ ml^−1^ every 24 h (grey lines). Error bars, ± one standard deviation. E. Growth analysis of three independent RNAi strains during knock-down of each *HAT* (target regions indicated in A). Un-induced, open circles; RNAi induced (+Tet), closed circles. Cells were split back to 1 × 10^5^ ml^−1^ every 24 h (grey lines). Error bars, ± one standard deviation. F. Northern analysis during RNAi knock-down. Membranes were probed with *HAT1* and *HAT2* gene fragments (see bars in A). Tubulin (*TUB*) was used as a loading control. Relative *HAT1* or *HAT2* mRNA signals, as determined by phosphorimager analysis and corrected for loading, are indicated. EtBr, ethidium bromide.

To examine MYST-family acetyltransferase function in bloodstream-form *T. brucei*, we initially attempted gene disruption or knockout (Fig. 4A). The *HAT* genes are ‘single copy’ and *T. brucei* is diploid, so we assembled a pair of constructs with different selectable markers for each gene. All six constructs targeted the correct loci as determined using PCR assays but we were unable to disrupt both alleles of either *HAT1* or *HAT2* (data not shown). In contrast, *HAT3* null strains were obtained and confirmed by Southern analysis (Fig. 4C). The results indicate that *HAT3* is dispensable and suggest that *HAT1* and *HAT2* are essential for growth. Although it was proposed that more abundant histone acetylation in lower eukaryotes reflects a higher proportion of transcriptionally competent chromatin, HAT3 knockout suggests that 73% H4K4 acetylation (Mandava *et al*., 2007; Siegel *et al*., 2008) has little impact on transcription.

We next generated conditional deficient strains for HAT1 and HAT2. We were able to disrupt both native alleles in *T. brucei* conditionally expressing ^GFP^HAT1 or ^GFP^HAT2 (Fig. 4B and see Fig. 3) indicating that both ^GFP^HAT1 and ^GFP^HAT2 complement the HAT defects. Downregulation of ^GFP^HAT1 and ^GFP^HAT2 expression produced a severe growth defect in both cases (Fig. 4D). In contrast, *hat3* null mutants grew at the same rate as wild type (Fig. 4D). This verified our assignment of HAT1 and HAT2 as essential for growth but the conditional growth defects were not stable. Phenotype instability was found to be due to the outgrowth of cells in which intact native HAT alleles were regenerated through recombination between the ectopic gene and portions of the coding region that remained intact in these strains (see Fig. 4A, data not shown). We therefore turned to RNA interference (RNAi) to generate stable knock-down strains. We used an RNAi system for conditional expression of long, intramolecular, ‘stem-loop’ (Durand-Dubief *et al*., 2003) double-stranded RNA (dsRNA) (517, 459 and 568 bp for HAT1–3 respectively) checked for specificity (Redmond *et al*., 2003). Expression of dsRNA specific for either *HAT1* or *HAT2* induced growth defects similar to those observed following downregulation of ^GFP^HAT1 and ^GFP^HAT2 (compare Fig. 4D and E). Northern analysis indicated specific knock-down of *HAT1* and *HAT2* mRNA respectively (Fig. 4F) and the growth defects following RNAi induction were highly reproducible in multiple independent strains (Fig. 4E) and at multiple times after prolonged culture (data not shown). *HAT3* RNAi strains provided controls for Tet induction in cells harbouring the RNAi vector system and, as expected, showed no growth defect (Fig. 4E).

### Cell cycle analysis following HAT1 or HAT2 knock-down

The histone H4 acetyltransferase, Esa1, is required for G_2_/M progression and is the only essential MYST-type HAT in *S. cerevisiae* (Clarke *et al*., 1999). In addition, the *T. brucei* deacetylase, DAC4, is required for normal G_2_/M progression (Ingram and Horn, 2002). No method is available to synchronize bloodstream-form *T. brucei*, but nuclear and mitochondrial (kinetoplast) DNA, stained with DAPI, provides excellent cytological markers that define position in the cell cycle (Woodward and Gull, 1990). During normal growth, approximately 80% of cells display a single nucleus and a single kinetoplast (1N1K) corresponding to earlier phases of the cell cycle (G_1_/S). A single nucleus and two kinetoplasts (1N2K) correspond to nuclear G_2_ and two nuclei and two kinetoplasts (2N2K) indicate completion of mitosis.

**Fig. 5:**
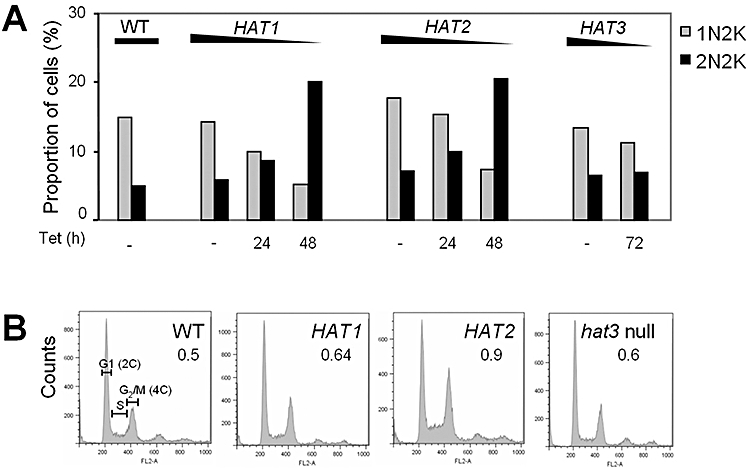
Cell cycle analysis following *HAT* knock-down. A. Analysis of DAPI-stained *T. brucei*. 1N2K indicates cells with a single nucleus and two kinetoplasts and corresponds to nuclear G_2_. 2N2K indicates cells with two nuclei and two kinetoplasts and corresponds to completion of mitosis. *n* > 300 cells for each sample per time point. B. DNA content analysis using flow cytometry. *HAT1*- and *HAT2*-depleted cells were analysed following 48 h +Tet. A *hat3* null strain was analysed in parallel. 2C, cells with a diploid nuclear content; 4C, cells that have passed through S-phase and replicated their DNA. The proportion of 4C relative to 2C is indicated. *n* = 20 000 cells.

We used the *HAT* RNAi strains to explore the role of MYST-type HATs in cell cycle progression in *T. brucei*. After 24 h of RNAi induction, we saw very little or no growth defect compared with un-induced strains. Between 24 and 48 h of induction, the *HAT1* and *HAT2* knock-down strains showed approximately five- and twofold reduction in cell density respectively (Fig. 4E). To examine primary rather than secondary defects, we analysed cell cycle position at 24 and 48 h, but focused on trends established by 24 h, prior to detectable growth defect. Within the G_2_/M population, in both *HAT1* and *HAT2* knock-down strains, we saw a reduction in the number of pre-mitotic (1N) cells and an increase in the number of post-mitotic (2N) cells (Fig. 5A) indicating accumulation pre-cytokinesis. A *HAT3* RNAi strain provided a control as neither this (Fig. 5A) nor a *hat3* null strain (data not shown) displayed evidence of cell cycle aberration.

We next analysed cellular DNA content by flow cytometry (Fig. 5B). A *hat3* null strain was not significantly different from wild type and, consistent with accumulation pre-cytokinesis, we saw a substantial increase in 4C (4× haploid nuclear DNA content) cells following *HAT2* knock-down. Interestingly, a similar increase in 4C cells was not seen following *HAT1* knock-down. DNA synthesis is normally complete before mitosis but the increased number of 2N2K cells in the absence of a corresponding increase in 4C cells indicates progression through mitosis without nuclear DNA replication.

### HAT1 modulates telomeric silencing

**Fig. 6:**
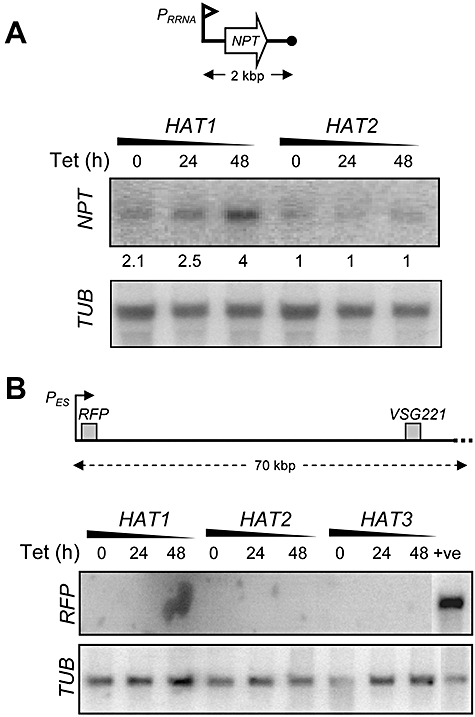
HAT1 modulates telomeric silencing but not *VSG* expression site repression. A. A ribosomal RNA promoter (*P*_*RRNA*_) driving the expression of an *NPT* gene was placed 2 kb from a telomere in *HAT* RNAi strains. *NPT* expression was assessed before and after knock-down by Northern blot. *TUB* was used as a loading control. Relative *NPT* expression, determined by phosphorimager analysis and corrected for loading, is indicated. B. *HAT1–3* knock-down was induced in strains with an *RFP* reporter immediately downstream of a repressed *VSG221* expression site promoter (*P*_*ES*_). *RFP* expression was assessed before and after knock-down using Northern blotting. The positive control is a similar strain but expressing *VSG221*. Tubulin (*TUB*) was used as a loading control. All of the knock-down samples expressed *RFP* at < 1% relative to the control as determined by phosphorimager analysis.

Many MYST-family HATs regulate transcription. In *T. brucei*, pol I transcription is repressed immediately adjacent to telomeres (Glover and Horn, 2006) in a telomere- (Glover *et al*., 2007) and sirtuin deacetylase- (Alsford *et al*., 2007) dependent manner. In *S. cerevisiae*, telomeric silencing is similarly dependent on a sirtuin and also the MYST-family HATs, Sas2 (Reifsnyder *et al*., 1996) and Esa1 (Clarke *et al*., 2006). The *S. pombe* MYST-family HAT mst2+ appears to perform a similar function (Gomez *et al*., 2005). To explore the role of MYST-HATs in telomeric silencing in *T. brucei*, we placed a reporter cassette driven by a ribosomal RNA promoter within 2 kb of a *de novo* telomere in the *HAT* RNAi strains (Fig. 6A); the reporter is repressed by telomere position effect in these strains. We first analysed *NPT* reporter expression before and after *HAT* knock-down by Western blotting with α-NPT. The analysis indicated increased telomeric reporter expression following *HAT1* knock-down with no change in expression following *HAT2* or *HAT3* knock-down (data not shown). To confirm these results, we analysed *NPT* reporter mRNA by Northern blotting before and after *HAT1* and *HAT2* knock-down (Fig. 6A). The analysis confirmed increased reporter expression following *HAT1* knock-down (twofold compared with un-induced control and fourfold compared with *HAT2* control) with no change in reporter expression following *HAT2* knock-down. Increased reporter expression was not simply due to loss of viability, because extracts prepared from the *HAT2* RNAi strain (Fig. 6A) and a histone variant RNAi strain (data not shown), which also suffered substantial loss of viability, had unchanged levels of *NPT* expression. Thus, telomeric silencing is specifically compromised following *T. brucei* HAT1 knock-down.

Most genes appear to be constitutively transcribed by RNA pol II in *T. brucei*, but antigenic variation, unusually, relies on regulated pol I transcription. Although multiple variant surface glycoprotein expression site promoters are found close to telomeres, all are repressed but one in the bloodstream form. These promoters are usually approximately 50 kb from telomeres and recruit RNA pol I despite being distinct in sequence compared with the ribosomal RNA promoter. We therefore examined the expression of a reporter placed immediately downstream of a repressed expression site promoter (Fig. 6B) following *HAT* knock-down. None of the HATs appear to play a primary role in expression site promoter repression as determined by Northern blot analysis of reporter expression (Fig. 6B). The data are consistent with previous reports of mechanistically distinct *VSG* expression site promoter silencing and telomeric silencing (Alsford *et al*., 2007; Hughes *et al*., 2007).

### HAT2 acetylates histone H4K10

**Fig. 7:**
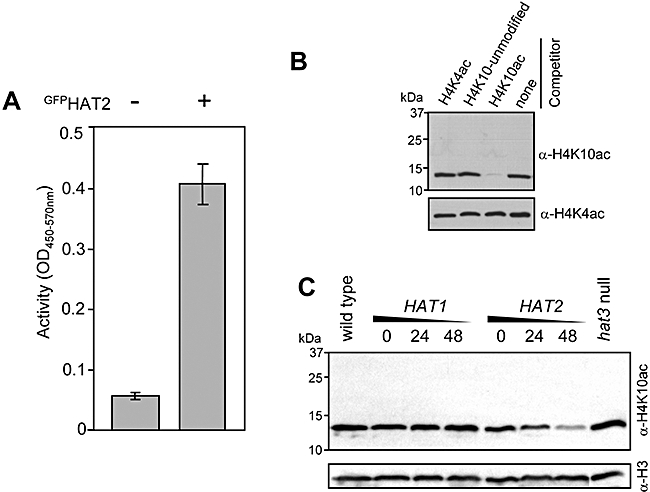
HAT2 acetylates histone H4K10. A. *In vitro* acetyltransferase activity assay using eluates from *T. brucei* lacking (−) or expressing (+) ^GFP^HAT2. The substrate was a *T. brucei* histone H4 tail peptide (H4^A1-L20^). *n* = 3. Error bars, ± one standard deviation. B. Characterization of α-histone H4K10ac. A Western blot of whole trypanosome extracts (2 × 10^6^ cells lane^−1^) was probed using α-H4K10ac. Peptide competitors used are shown above. To confirm equal loading, the blot was stripped and reprobed with α-H4K4ac (Siegel *et al*., 2008). C. Specific depletion of the H4K10ac signal following HAT2 knock-down by RNAi. Western blotting was carried out as for (B) but without peptide competitor. Note that Siegel *et al*. (2008) demonstrated that total H4 levels are not depleted during HAT2 knock-down. To confirm equal loading, the blot was stripped and reprobed with α-histone H3.

The key residues involved in MYST factor catalysis are Glu^338^ and Cys^304^ (Yan *et al*., 2002) but motif A and the C_2_HC motif are also conserved and involved in Ac-CoA binding (Sternglanz and Schindelin, 1999) and HAT activity in some MYST-family proteins respectively (Takechi and Nakayama, 1999; Akhtar and Becker, 2001). Both motif A and the C_2_HC motif are absent from all three trypanosomatid HAT2s but neither motif is absolutely required for activity as demonstrated by Esa1 which complements *esa1* cell cycle defects when motif A is mutated (Smith *et al*., 1998) and displays a TFIIIA-type zinc finger fold in place of the C_2_HC motif (Yan *et al*., 2000). To determine whether HAT2 displays acetyltransferase activity, we first asked whether the protein could transfer acetyl groups to a *T. brucei* histone H4 peptide substrate *in vitro*. As several recombinant MYST-family acetyltransferases lack activity in the absence of cofactors (Iizuka and Stillman, 1999; Sutton *et al*., 2003), we used ^GFP^HAT2 purified from *T. brucei* for this analysis (see Fig. 3). Importantly, we previously demonstrated that ^GFP^HAT2 complements the *hat2* defect (Fig. 4E) indicating association with any additional factors required for activity. Despite the absence of a canonical motif A or C_2_HC motif within HAT2, affinity-purified ^GFP^HAT2 (complex) displays acetyltransferase activity *in vitro* (Fig. 7A). We also assembled a mutant ^*GFP*^*HAT2(C351A)* (see Fig. 2) ORF but the protein was expressed at a level insufficient for an *in vitro* assay, possibly due to selection against dominant-negative replacement of native HAT2 complexes (data not shown). This, and the finding that ESA1(E338Q) was dominant negative (Yan *et al*., 2000), is consistent with the hypothesis that HAT activity is essential for MYST function.

Importantly, acetylation of a *T. brucei* histone H4 tail peptide does not necessarily reflect the nature of the substrate *in vivo* as substrate specificity can be lost or compromised *in vitro*. As substrate specificity underpins the epigenetic code, we sought to link acetyl modifications to specific HATs *in vivo*. The highly divergent sequence of the trypanosome histone H4 N-terminal tail prohibits the use of commercially available antibodies to specific modifications but *T. brucei* histone-specific antibodies were recently used to demonstrate that HAT3, but not HAT1 or HAT2, acetylates H4K4 (Siegel *et al*., 2008). A similar approach was used to generate antibodies to acetylated H4K10 and specificity was tested by pre-incubation with peptide competitors before Western blotting. This demonstrated α-H4K10ac affinity only for the corresponding peptide and no cross-reactivity to other modified or unmodified sites (Fig. 7B). We then used α-H4K10 to screen a series of *T. brucei* protein extracts harvested from wild-type, *HAT1* and *HAT2* RNAi strains induced for different periods and a *hat3* null strain. This demonstrated a reduction in signal specific to the *HAT2* knock-down strain (Fig. 7C). We conclude that trypanosomes use distinct MYST-family acetyltransferases to acetylate K4 and K10 on the N-terminal tail of histone H4; H4K4 (73% of sites modified, see above) is acetylated by the dispensable HAT3 while H4K10 (7%) is acetylated by the essential HAT2. The data also show that loss of H4K4 acetylation has no discernible effect on H4K10 acetylation (Fig. 7C) and vice versa (Siegel *et al*., 2008).

## Discussion

Histone modification is under study in a range of parasitic protozoa (Ramakrishnan *et al*., 2004; Smith *et al*., 2005; Merrick and Duraisingh, 2006; Sullivan *et al*., 2006). Histone acetylation and methylation have been described in *T. brucei* (Janzen *et al*., 2006a) and *T. cruzi* (da Cunha *et al*., 2006) and it is thought that trypanosomatids employ a relatively simple histone code to regulate access to DNA (Horn, 2007). *T. brucei* expresses three distinct MYST-family members, all of which have homologues in *T. cruzi* and *Leishmania* and we describe non-redundant roles for each of these histone acetyltransferases in bloodstream-form *T. brucei*. HAT1 modulates telomeric silencing and is required for growth, and possibly, for DNA replication; HAT2 is required for H4K10 acetylation and growth; and, HAT3 is required for H4K4 acetylation and is dispensable for growth. The orthologues in other trypanosomatids likely have similar roles. The non-redundant functions for *T. brucei* HAT1–3 appear to reflect unique substrates for each acetyltransferase and further support the idea of a simplified, non-redundant histone code in these divergent parasites.

Both HAT1 and HAT2 are essential for growth in bloodstream-form *T. brucei*, as demonstrated using both conditional expression in a null background and knock-down using RNAi. To our knowledge, this is the first example of a unicellular eukaryote with two essential MYST-family acetyltransferases. For comparison, *S. cerevisiae* (Howe *et al*., 2001) and *S. pombe* (Gomez *et al*., 2005) each express a single essential MYST acetyltransferase. Esa1 is essential in *S. cerevisiae* (Smith *et al*., 1998; Clarke *et al*., 1999) while the other, non-essential, MYST-family members, Sas2 and Sas3, also function in transcription regulation and cell cycle control (Reifsnyder *et al*., 1996). Interestingly, *S. cerevisiae* Sas3 displays redundancy with Gcn5, a non-MYST acetyltransferase. In the absence of Gcn5, Sas3 is also essential (Howe *et al*., 2001). This is thought to be because Esa1 favours histone H4 as substrate while Sas3 and Gcn5 both favour histone H3. Thus, two MYST-family acetyltransferases may be essential in *T. brucei* due to the absence of a Gcn5 homologue (see table S4 in Ivens *et al*., 2005). Further work will be required to determine whether the MYST-family HATs acetylate histone H3 lysine residues or other additional sites in *T. brucei*.

In *S. cerevisiae*, histone acetylation is essential for cell cycle progression (Clarke *et al*., 1999), and cells lacking either histone H3 or H4 tails are unable to progress through G_2_/M (Ling *et al*., 1996). Some conventional cell cycle checkpoints may be absent from *T. brucei* (Hammarton, 2007) but the *T. brucei* deacetylase, DAC4, is required for normal G_2_/M cell cycle progression (Ingram and Horn, 2002). Cells accumulate pre-cytokinesis following *HAT1* or *HAT2* knock-down and the absence of a parallel increase in tetraploid cells may reflect a DNA replication defect upon *HAT1* knock-down. Indeed, yeast Sas2 is involved in chromatin assembly associated with DNA replication (Meijsing and Ehrenhofer-Murray, 2001; Osada *et al*., 2001). A similar defect was reported in cells defective in H3K79 methylation but in that case the cells progressed through cytokinesis to produce cells with a haploid DNA content (Janzen *et al*., 2006b).

Histone acetylation is predominantly linked to increased transcription as opposed to repression and can increase transcription by all three major eukaryotic RNA polymerases (Ura *et al*., 1997; Hirschler-Laszkiewicz *et al*., 2001). Acetylation may influence transcription by altering promoter accessibility or by facilitating elongation. The MYST complexes tend to exert their effects over domains rather than by targeting promoters. Most genes are constitutively transcribed by RNA pol II in *T. brucei* and conventional pol II promoters have not been identified for protein-coding genes (Palenchar and Bellofatto, 2006). Unusually, in *T. brucei*, the variant surface glycoprotein genes are transcribed by RNA pol I and regulation here appears to be at the level of transcription elongation (Vanhamme *et al*., 2000) while transcription mediated by the canonical ribosomal RNA pol I promoter is repressed immediately adjacent to telomeres in a sirtuin (SIR2rp1) deacetylase-dependent manner (Alsford *et al*., 2007). We demonstrate that HAT1 modulates telomeric silencing in *T. brucei* but neither SIR2rp1 (Alsford *et al*., 2007) nor HAT1–3 (this study) appear to have a role in variant surface glycoprotein expression site repression. The role in telomeric silencing is reminiscent of *S. cerevisiae* Sas2 (Reifsnyder *et al*., 1996) and Esa1 (Clarke *et al*., 2006) and *S. pombe* mst2+ function (Gomez *et al*., 2005). Sas2 is a histone H4K16-specific acetyltransferase (Meijsing and Ehrenhofer-Murray, 2001; Sutton *et al*., 2003; Shia *et al*., 2005) that opposes the action of the Sir2 histone deacetylase to establish boundaries between telomeric heterochromatin and euchromatin (Kimura *et al*., 2002; Suka *et al*., 2002). Deletion of Sas2 allows Sir2-dependent heterochromatin to extend further into subtelomeric DNA (Kristjuhan *et al*., 2003) and this redistribution of a limiting pool of Sir2 dilutes the effect immediately adjacent to the telomere. Our results therefore are consistent with a conserved role in telomeric silencing from yeast to trypanosomatids. Other MYST-family acetyltransferases have been shown to be required for repression but these effects are also now thought to be indirect. For example, Sas3 was originally identified as a regulator of gene silencing (Reifsnyder *et al*., 1996), but is now thought to stimulate transcription and replication (John *et al*., 2000). We cannot rule out a direct role for acetylation in telomeric silencing (Braunstein *et al*., 1996) but, like Sas2/Esa1 and Sir2 in *S. cerevisiae*, we propose that HAT1 and the sole nuclear sirtuin deacetylase, SIR2rp1, establish boundaries between transcriptionally active and repressed telomeric domains in *T. brucei*.

Although histone N-terminal sequences are highly divergent in trypanosomes, a number of specific lysine residues are acetylated within the N-terminal tail of *T. brucei* histone H4 (Mandava *et al*., 2007). Antisera against human and yeast histone modifications do not recognize specific modification on *T. brucei* histones so specific antibodies against trypanosome modifications are required to facilitate both identification of the relevant modifiers and further studies on HAT function. Specific antibodies have been used to show that HAT3 acetylates H4K4 (Siegel *et al*., 2008) and we now show that HAT2 acetylates H4K10. In humans (Sobel *et al*., 1995), flies (Sobel *et al*., 1994) and *Tetrahymena* (Chicoine *et al*., 1986), histone H4K5 and H4K12 are diacetylated by a cytosolic type-B HAT (Parthun *et al*., 1996). The role of these modifications is poorly understood but they may function in histone deposition (Sobel *et al*., 1995). Trypanosome H4K4, acetylated at > 70% of sites, does not appear to be analogous to H4K5 in other eukaryotes (Siegel *et al*., 2008) raising the possibility that trypanosome H4K5 and K10, each acetylated at about 7% of sites (Mandava *et al*., 2007), could be analogous to H4K5 and H4K12. Whatever the precise function of H4K4ac and H4K10ac, the presence of a pair of residues within the first 10 N-terminal residues of histone H4 independently targeted by specific MYST-family proteins is unprecedented and has implications for the evolution of the epigenetic code and for the interpretation of the code in different organisms.

Trypanosomatid genomes encode relatively few acetyltransferases, methyltransferases and cognate binding modules (see table S4 in Ivens *et al*., 2005), and the histones also display fewer modifications (da Cunha *et al*., 2006; Janzen *et al*., 2006a; Mandava *et al*., 2007), compared with other eukaryotes amenable to experimental analysis. Low-level redundancy should facilitate further functional dissection of the processes enacted by the *T. brucei* acetyltransferases.

## Experimental procedures

### *T. brucei* growth and manipulation

All strains were derived from Lister 427 bloodstream-form MITat1.2 (clone 221a). *T. brucei* were grown in HMI-11 and transformed with linear DNA constructs as previously described (Alsford *et al*., 2005). For expression of tagged proteins, and for RNAi, recombinant vectors were integrated at a marked *RRNA* locus in 2T1 *T. brucei* that express the tetracycline repressor (TetR) (Alsford *et al*., 2005). Drugs were added ∼6 h post transfection. Recombinant protein expression and RNAi were induced by growing *T. brucei* in 1 μg ml^−1^ tetracycline (Sigma) for 24 h unless stated otherwise. For growth analysis, *T. brucei* were seeded at a density of 1 × 10^5^ ml^−1^ and split back to 1 × 10^5^ ml^−1^ every 24 h where necessary. Counts were carried out using a haemocytometer.

### Plasmid constructs

DNA fragments were amplified by PCR from genomic DNA using Phusion high-fidelity DNA polymerase (New England Biolabs) and *T. brucei* Lister 427 genomic DNA in conjunction with specific primer pairs (relevant restriction enzyme sites are indicated in italics and stop codons in lower-case below). For N-terminal eGFP-tagging constructs, p^GFP^HAT1–3, we used the Tet-responsive expression vector pRPa^GFP^ (Alsford *et al*., 2005) and primers were as follows: for HAT1, HAT15N (GC*TCTAGA*GTAATGTTTGAGGTGCGGC) and HAT13N (GA*AGATCT*ttaTACTTTCTCAGCTCTACG); for HAT2, HAT25N (GC*TCTAGA*GCGTCGTTAGCAGCAAAAAA) and HAT23N (CG*GGATCC*tcaGCTCTGGTGCTGATTG); and for HAT3, H35N (GC*TCTAGA*AAACGACAGAGGTCGGGA) and H33N (CG*GGATCC*ttaTGATATCGGCACCCATAG).

For *HAT1* and *HAT2* gene disruption constructs, we amplified and cloned DNA fragments using the following primer pairs: for *HAT1*, 29M45 (TCC*CCGCGG*TCCTGCGACGTTAGGACTTA) and 29M43 (GG*GGTACC*GGTGTAGTCTTCTCTTTG) and for *HAT2*, 18M145 (TCC*CCGCGG*TACTATGCGCCTTATGGTA) and 18M143 (GG*GGTACC*GAATAGAAGAGTGAGGTAG). A 130 bp BamHI–Bsp120I fragment from *HAT1* and a 679 bp EcoRV–SmaI fragment from *HAT2* were replaced by blasticidin S deaminase (*BSD*) or puromycin *N*-acetyltransferase (*PAC*) selectable marker gene cassettes. For *HAT3* knockout we amplified upstream and downstream targets using the H31 (CC*ACTAGT*ACCCCAGTAGA), H32 (ATC*CTGCAG*TCCACCTATACGGAAGGGA) and H33 (GG*GGTACC*AGTGTTTTTCACCTGTTT), H34 (CT*GGTACC*TCAGAAACAGG) primer pairs respectively. The targets were assembled such that they flanked a *PAC* or neomycin phospotransferase (*NPT*) selectable marker.

We used a stem-loop vector (pRPa^iSL^) to generate intramolecular dsRNA for RNAi. The RNAi trigger fragments were amplified using the following primers: for *HAT1*, H1R5SL (GATC*GGGCCCGGTACC*CCCTTTTCGGAACATGAAGA) and H1R3SL (GATC*TCTAGAGGATCC*AGTGTGACGCAGTGACTT); for *HAT2*, H2R5SL (GATC*GGGCCCGGTACC*ACTCACAGACTTGGGAGCA) and H2R3SL (GATC*TCTAGAGGATCC*TCATGTCATGTGCCCACTTT); and for *HAT3*, H3R5SL (GATC*GGGCCCGGTACC*TGACCTCATTAAGCACGCTG) and H3R3SL (GATC*TCTAGAGGATCC*AAGGGCAAGGGCAGTATCTT).

### DNA and RNA analysis

PCR, RT-PCR, Southern and Northern analysis were all carried out according to standard protocols. Signals on Northern blots were quantified using a Phosphorimager (Amersham).

### Protein analysis

SDS-polyacrylamide gel electrophoresis and Western blotting were performed according to standard protocols (Ausubel *et al*., 1998). Western blots were developed using rabbit α-GFP RF795PB (kind gift from M. Rout) or rabbit α-GFP 11E5 (Molecular Probes) according to the manufacturer's instructions and tagged proteins were detected using enhanced chemiluminescence (Amersham). Western blot analysis using α-H4K10ac and α-H3 (Abcam, 1791) were carried out as described (Siegel *et al*., 2008). Indirect immunofluorescence microscopy was carried out as described (Alsford *et al*., 2007).

### Cell cycle analysis and flow cytometry

Nuclear and kinetoplast DNA, counterstained with DAPI, were analysed as described (Ingram and Horn, 2002). For flow cytometry, 1 × 10^6^ cells were fixed in 70% methanol, 30% PBS and incubated at 4°C overnight. Cells were washed in PBS then re-suspended in PBS containing 10 μg ml^−1^ propidium iodide and 10 μg ml^−1^ RNase A and incubated at 37°C for 45 min. A Becton Dickinson FACScalibur was used to analyse samples with cell quest software and detector FL2-A with an Amp gain value of 1.75. Data analysis was carried out using FlowJo (Treestar).

### Histone acetyltransferase assay

Cells (1.5 × 10^8^) expressing ^GFP^HAT2 or the wild-type strain were lysed in RIPA buffer (Upstate) containing protease inhibitor cocktail (Sigma) at 4°C. Tagged protein (complex) was enriched using Catch and Release columns (Upstate) according to the manufacturers' instructions. Briefly, 9 μl of rabbit α-GFP serum (M. Rout, Rockefeller University) and 430 μl of lysate were applied to each column and incubated for 6 h at 4°C with continuous rotation followed by washing and elution with 70 μl of 1× non-denaturing buffer. An indirect ELISA (Upstate) was used to measure HAT activity according to the manufacturers' instructions. Briefly, streptavidin-coated plates were loaded with 1 μg ml^−1^*T. brucei* histone H4 peptide (H4^A1-L20^), biotin conjugate. Eluate (20 μl) was added followed by incubation at 30°C for 30 min followed by incubation with rabbit α-Ac-Lys and goat α-rabbit HRP conjugate both at 0.4 ng ml^−1^. Signals were quantified on a plate reader at 450 and 570 nm.
